# Contradiction between Supply and Demand of Public Sports Services and Coping Strategies Based on the Genetic Algorithm

**DOI:** 10.1155/2022/1227981

**Published:** 2022-09-05

**Authors:** Liu Lu, Wei Wei

**Affiliations:** ^1^College of Physical Education, Chengdu Sport University, Chengdu 610041, China; ^2^College of Sports Training, Chengdu Sport University, Chengdu 610041, China

## Abstract

The current situation of China's sports public service is not very satisfactory. This research analyzes not only the operation characteristics of the public sports service supply mode but also the connotation mechanism of the public sports service collaborative supply. The article also constructs the mechanism elements and the index system of the public sports service collaborative supply from the perspective of the genetic algorithm. Taking the eastern and central regions as research cases, this paper analyzes the actual situation of the collaborative supply of public sports services, and further explores the gap between urban and rural public sports. It puts forward the ideas and strategic paths for the innovative development of the collaborative supply model of public sports services. This research uses descriptive statistical analysis on the evaluation results of public sports service quality to examine the actual performance value of public sports service quality in various regions and uses the characteristics of different fitness of genetic algorithms to select the optimal supply and demand of public sports services in different regions. By analyzing the characteristics of insufficient supply and demand of public sports services in various regions, an optimal genetic algorithm model is constructed, and then combined with the scope of the study area and the number of people in the area, the genetic algorithm is used to continuously optimize until a set of optimal solutions appears, thus completing the optimal configuration of public sports services. The optimal path for the development of public sports in different regions is obtained, so as to promote the development of regional public sports. The study found that the satisfaction of sports funds in primary and middle schools in the east and middle is not very high, and the satisfaction with sports funds in middle schools is higher than that in primary schools, but only 50% of schools choose that sports funds are basically sufficient. The difference in satisfaction with sports funding in middle schools in the eastern and central regions is not obvious, and the satisfaction with sports funding in primary schools in the eastern region is higher than that in the central region. Judging from the amount of sports expenditures filled in by schools, 2000–3000 yuan per school year for primary schools and 4000–5000 yuan for middle schools are more common. Based on the calculation of 1,000 students in a school, the per capita sports expenditure is less than 10 yuan, which is very limited for sports training. This research will help to change the service concept and scientific decision-making and change the supply mode of sports public services. It will establish not only a vertical, complete, and horizontally smooth organizational structure and coordination mechanism but also a clear strategy for equalization of urban and rural sports public services.

## 1. Introduction

At present, the supply mode of urban public sports service in China is still in the initial stage of development. Therefore, many sports resources are difficult to play their potential role and cannot play their due role in improving the health and quality of life of the general public. The specific problems of the supply of public sports facilities include insufficient supply of public sports facilities, single type, untimely community maintenance and management, low spatial combination, unbalanced and unreasonable layout, as well as insufficient number of social sports instructors. It is expected to promote the market-oriented operation process of China's public sports service supply, so as to further optimize the allocation of urban public sports resources and realize the efficient and practical use of public sports resources. Under the background of service-oriented government, the focus of China's sports development is to promote the participation of all people in sports and improve the public sports service system. The purpose of this paper is to provide a basis for determining the supply goals and contents of public sports services in the future and to make certain references for the improvement of the national fitness program, sports functional departments, and social security service system.

Achieving the equalization of public sports services will help promote the development of sports in China. Xiangwei believed that the development of the city drives the progress of the entire economy and society. Meanwhile, the development of urban leisure sports has become an important engine of modernization [1]. Sun and Hu believed that people's average life expectancy has increased [2]. Chung aimed to elucidate how the organizational commitment and service orientation of sports practitioners are related to their job performance, with a view of helping sports practitioners provide good service and effectively organize their formation [3]. He believes that it is very important to promote comprehensive agricultural improvement [4]. Li believed that the services are a key link in the reform of the public service system [5]. Their proposed application of public sports services to the contradiction between supply and demand is not very effective for this situation. Through referring to the previous data, a genetic algorithm to optimize public sports services is proposed.

In the process of practice and application of the genetic algorithm, due to the limitation of population size and other factors, some excellent individuals will reproduce prematurely in the process of its evolution, thus reducing the diversity of the population. Tavakkoli-Moghaddam et al. proposed a GA algorithm [6]. Volkanovski A proposed a new method [7]. Long and Wu studied global optimization [8]. Gong et al. proposed an ensemble-based genetic algorithm [9]. Nemati et al. proposed an improved MILP method [10]. In the process of rapid industrialization, the agglomeration of population to cities has become the main source of “urban diseases,” and community public sports services are facing severe challenges. Genetic algorithm has a better way of dealing with combinatorial optimization problems because it has better robustness, and the genetic algorithm will be further explored later.

The effective supply of public sports facilities is not only an important guarantee for the steady implementation of the national fitness strategy but also a powerful tool to solve the main social contradictions in the new era. This paper investigates the supply and demand of public sports services based on the genetic algorithm and finds out the problems existing in its supply based on the background of supply-side reform. The article not only analyzes the reasons for supply imbalance but also discusses the factors that lead to supply imbalance, as well as puts forward countermeasures and suggestions for supply-side structural reform. Starting from the purpose of building a service-oriented government, through interviews, comparative analysis, literature, field investigation, and mathematical statistics, the current situation of public sports service supply, service consultation, facility construction, capital investment, activity development, and other issues and their effects are studied. Data collection and analysis are also carried out. The residents' public sports survey found that among the first, fitness paths accounted for 67%, far exceeding other sports venues. In the second place, outdoor fitness equipment and parks and squares accounted for 44%, which is the second main choice for residents to participate in sports activities.

## 2. Contradiction between Supply and Demand of Public Sports Services and Coping Strategies

### 2.1. Public Sports Services and Supply and Demand Equilibrium Theory

#### 2.1.1. Public Sports Services

With the continuous development of society and the continuous growth of sports organizations, the role of sports activities in people's lives is becoming increasingly important, which plays a significant role in promoting people's health and improving people's quality of life. The government's single supply method is difficult to meet the needs of the general public in terms of public sports services, and it also makes many public sports resources idle and wasteful, making it difficult to give full play to the potential use value of public sports resources, which fails to achieve the original intention of serving the people. Under this situation, the government relaxes its powers and allows more social forces to enter the public sports service undertaking, which in turn produces a variety of public sports service supply methods. In particular, the market-oriented way is the most prominent, and it is also the most effective way of supplying public sports services. The market-oriented operation mode of China's public sports service supply is a reform of the traditional supply mode of China's public sports service. The reform is not simply the release and transfer of the government's power to the market, but the orientation of market-oriented reform as the core. With competition and monopoly, relying on market organizations and non-governmental organizations to achieve the diversification and efficiency of China's public sports service supply, only in this way it can move towards the marketization of public sports service supply [11].

The market-oriented operation of public sports service supply is the process of various activities to achieve the goal of public sports service supply. In this supply-demand relationship, the relevant government departments are the suppliers of it, and the masses are the demanders of it. The diversification of supply subjects enables the supply structure to be adjusted, which is also consistent with the supply-side structural reform. Meanwhile, as a supply product, public sports services are also diversified, such as public sports facilities, public sports information guidance, and public sports personnel guidance. Adjusting the structure of different types of public sports products also belongs to the category of supply-side structural reform. Strictly speaking, public sports service is the basic responsibility of the sports departments of governments at all levels, and it is the basis for implementing the outline of the national fitness plan and carrying out various activities of national fitness. Only by continuously increasing the construction of public sports services can it have the conditions to truly implement the outline of the national fitness plan, so that increase in physical activity in China can be realized and the growing public sports needs of the masses can be met [12].

#### 2.1.2. Supply and Demand Equilibrium Theory

As a public sector, the government has the function of supplying those public goods that the market cannot provide or provide insufficiently, so as to achieve the purpose of making up for market defects. The characteristic of public goods is that every citizen of the society has the right to obtain it. The subject of its demand is the citizen, and its total demand is the sum of the demand of social citizens. When the public goods supplied by the government and the public goods demand of citizens are roughly in line with each other, the supply of public goods by the government and the public goods demand of citizens reach a state of structural equilibrium, but the structural equilibrium achieved is generally temporary and relative. In many cases, the public products provided by the government are not what citizens need, while the public products that citizens need are often not provided by the government; that is, supply and demand are in an unbalanced state. Even if it is an unbalanced state, it is limited by the scope of time and space, and it is an imbalance in line with the equilibrium theory of supply and demand [13].

### 2.2. Public Sports Service Supply System

**Figure 1:**
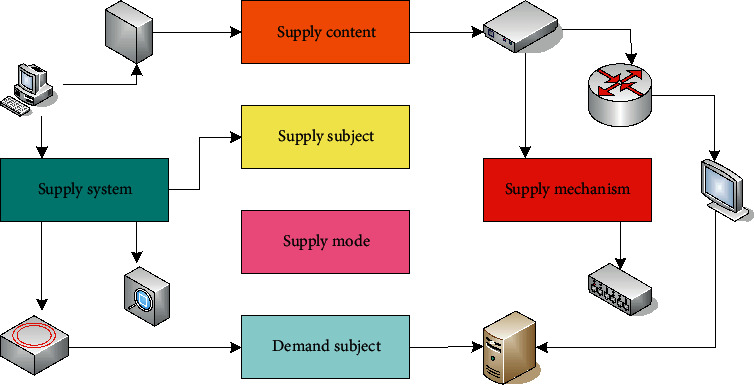
Composition of the public sports service supply system.

The composition of the public sports service supply system is shown in Figure 1. Providers generally refer to the fund providers of public sports products or public sports labor services, while producers generally refer to the specific executors of the production, processing, or public sports labor services of public sports products. When providing public sports services, there will be two situations in which the provider and the producer are the same or different. The situation where the provider is the same as the producer is called direct supply. Otherwise, it is called indirect supply.

**Figure 2:**
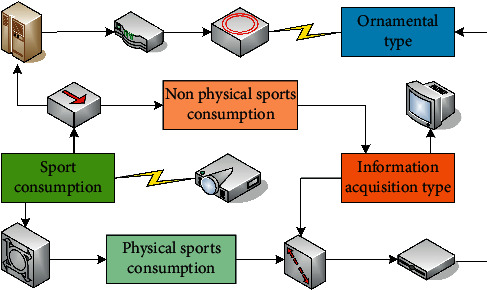
Sports consumption classification.

Sports consumption classification is shown in Figure 2. In the classification of sports consumption, physical and non-physical sports consumption is divided according to whether the purchased product has a physical form. Spectator-type sports consumption refers to the behavior that citizens purchase sports tickets, sports performance tickets, etc., by paying money for the purpose of satisfying their own sensory stimulation needs, such as watching sports games or sports performances on the spot. Information-acquisition-based sports consumption refers to the behavior of citizens to purchase sports guidance services through payment currency to meet the needs of health and fitness, such as paying for fitness guidance or consultation.

### 2.3. Genetic Algorithms

In the optimal allocation of the contradiction between supply and demand, the optimization process of the genetic algorithm is as follows: take the public sports service demand distributed to users in various regions as the decision variable, encode the decision variable to form the initial feasible solution set (i.e., generate several groups of initial public sports allocation schemes), and then substitute it into the constructed optimization model to eliminate and select by judging the advantages and disadvantages of each solution, so as to generate a new generation of feasible solution set, repeat the optimization process until a set of optimal solution sets appear, so as to complete the optimal allocation of public sports services.

This study uses the fitness characteristics of genetic algorithms to improve and optimize public sports services. Genetic algorithm is a cyclic process, and new individuals are continuously generated through crossover and mutation of individuals. However, because the operation is random, the individual with the best fitness will be destroyed. The efficiency of the algorithm will be negatively affected. In order to keep the best individual, the optimal individual is stored in a space through the optimal individual preservation strategy, and it is used to replace the individual with the lowest fitness in the calculation. This strategy ensures that the optimal individual is not destroyed by genetic operations and enables the optimal individual to spread rapidly in the population. Exploring the ideas and strategic paths of innovative development of the collaborative supply model of public sports services through genetic algorithms.

The objective equation is as the following formula:

(1) $$\begin{array}{c} Q=\sum_{i=1}^{N}wt_{i}\overset{K}{\underset{k=1}{\sum }}wt_{i,k}\times Q_{i,k}^{j}, \end{array}$$

where *wt*_*i*_ is the weight of the activity (i) in the project [14].

The crowding distance is as the following formula:

(2) $$\begin{array}{c} L{\left[s\right]}_{dis}=L{\left[s\right]}_{dis}+\frac{\left|L\left[s+1\right]m-L\left[s-1\right]m\right|}{L\left[k\right]m}. \end{array}$$

In formula (2), *L*[*s* − 1]*m* is the *m*th objective function value of the *s*th individual.

Assuming that starting from *t* = 0, *c* remains constant, then formula (3) can be obtained as

(3) $$\begin{array}{c} m\left(H,t+1\right)={\left(1+c\right)}^{i}m\left(H,0\right). \end{array}$$

The meaning of Cauchy distribution is that Cauchy distribution is a continuous probability distribution where mathematical expectation does not exist. When a random variable *X* satisfies its probability density function, *X* is said to obey the Cauchy distribution. The density function of the Cauchy distribution is as formulas (4) and (5):

(4) $$\begin{array}{c} f\left(x\right)=\frac{1}{\pi }\cdot \frac{t}{t^{2}+x^{2}},-\infty <x<\infty , \end{array}$$

(5) $$\begin{array}{c} F_{i}\left(x\right)=\frac{1}{2}+\frac{1}{\pi }\arctan\left(\frac{x}{t}\right). \end{array}$$

For a given population of size *n* in generation *t*, the general description is as formulas (6) and (7):

(6) $$\begin{array}{c} f_{\max}\left(t\right)=\max\left\{f\left(a_{1}\left(t\right)\right),f\left(a_{2}\left(t\right)\right),\ldots ,f\left(a_{n}\left(t\right)\right)\right\}, \end{array}$$

(7) $$\begin{array}{c} f_{\max}\left(t+1\right)=\max\left\{f\left(a_{1}\left(t+1\right)\right),f\left(a_{2}\left(t+1\right)\right),\ldots ,f\left(a_{n}\left(t+1\right)\right)\right\}. \end{array}$$

In dense blocks, the input of each layer is the output of the previous layer, namely,

(8) $$\begin{array}{c} x_{n}=H_{n}\left(\left[x_{0},x_{1},\ldots ,x_{n-1}\right]\right). \end{array}$$

Given a sample set that satisfies the independent and identical distribution, the task of the restricted Boltzmann machine is to find the value of the parameter *θ* = {*w*, *a*, *b*}, and then fit the given learning sample. Formula (9) can get through derivation of the maximum log-likelihood function [15]:

(9) $$\begin{array}{c} L\left(\theta \right)=\frac{1}{N}\sum_{n=1}^{N}Logp_{\theta }\left(v^{n}\right)-\frac{\lambda }{N}{\left\|W\right\|}^{2}. \end{array}$$

When *L* is the largest, the corresponding parameter *W* is as the following formula:

(10) $$\begin{array}{c} \frac{\partial L\left(\theta \right)}{\partial W_{i,j}}=Ep_{\text{data}}\left[v_{i}h_{j}\right]-Ep_{\theta }\left[v_{i}h_{j}\right]-\frac{2\lambda }{N}W_{ij}. \end{array}$$

Assuming that *L* represents the set of load balancing indicators, and the set *L*={*L*_1_, *L*_2_,…*L*_*n*_}, then *Lj*(*X*) represents the load indicator of cluster node *j* under the scheduling policy *X*. Then *Lj*(*X*) defines as the following formula:

(11) $$\begin{array}{c} L_{j}\left(X\right)=\frac{1}{T\left(X\right)}\sum_{i=1}^{m}M_{ij}*W_{ij}. \end{array}$$

Assuming that *ρ*(*A*) represents the error of the load index, then *ρ*(*X*) can be defined as the following formula:

(12) $$\begin{array}{c} \rho \left(X\right)=\sqrt{\frac{1}{n}{\left(L_{j}\left(X\right)-\frac{1}{n}\sum_{j=1}^{n}L_{j}\left(X\right)\right)}^{2}}. \end{array}$$

At time *t*, the pheromone concentration of server node *j* is initialized as the following formula:

(13) $$\begin{array}{c} \tau_{ij}\left(t\right)=\frac{1}{LF_{j}}. \end{array}$$

The pheromone (Pheromone, also known as pheromone, refers to the secretion of an individual into the body, and is detected by other individuals of the same species through the olfactory organs (such as the accessory olfactory bulb and the vomeronasal organ). It is the substance that causes the latter to exhibit some behavioral, emotional, psychological, or physiological change. Concentration changes simultaneously with the heuristic information. At time *t*, the heuristic information is initialized as the following formula:

(14) $$\begin{array}{c} n_{ij}\left(t\right)=\tau_{ij}\left(t\right). \end{array}$$

Pheromone update is defined as the following formula:

(15) $$\begin{array}{c} \tau_{ij}\left(t+1\right)=\left(1-\rho \right)\tau_{ij}\left(t\right)+\Delta \tau_{ij}\left(t,t+1\right). \end{array}$$

In formula (15), *ρ* represents the pheromone volatilization coefficient.

At time *t* + 1, the heuristic information changes as the following formula:

(16) $$\begin{array}{c} n_{ij}\left(t+1\right)=\tau_{ij}\left(t+1\right). \end{array}$$

The execution time of each resource needs to be calculated to execute the task using a matrix such as decoding sequence. Therefore, the total time to complete the resource scheduling task is as the following formula:

(17) $$\begin{array}{c} F\left(x\right)=\overset{n}{\underset{r=1}{\max}}\overset{W}{\underset{i=1}{\sum }}\text{work}\left(r,i\right). \end{array}$$

In formula (17), work (*r*, *i*) represents the time spent by resource *r* to execute subtask *i* on the resource, and *W* represents the number of subtasks assigned to the resource.

## 3. Research Objects and Method

### 3.1. Research Objects

The selection of the sample adopts the method of random classification sampling. The subordinate communities of each sub-district office are selected separately, and the specific selection number is 16 community managers and 320 community residents.

### 3.2. Interview Method

Interviews with relevant leaders of the National Sports Federation on the nature, management, operation, and funding sources of individual sports associations are conducted, and interviews with the relevant leaders of the Social Sports Section of the Sports Bureau on the construction of venues and equipment, management mechanisms, social sports instructors, and funding for community sports are also conducted. The development of community sports can be grasped and relevant information can be obtained. After communicating with the person in charge of the advanced sports unit on the organization and management of sports activities in the community, the source of funds, the participation of residents, the status of venue equipment, etc., first-hand information can be obtained. The research mainly adopts the combination of expert opinions and boundary delineation for index selection. The delineation of the threshold is to calculate the arithmetic mean (the arithmetic mean is the most basic and commonly used average index in statistics, and it is divided into simple arithmetic mean and weighted arithmetic mean), the frequency of full marks, and the coefficient of variation according to the selected scores of each indicator by experts. Each indicator has these three different discriminant scales.

The arithmetic mean calculation formula is as the following formula:

(18) $$\begin{array}{c} M_{j}=\frac{1}{n}\sum_{i=1}^{n}c_{ij}. \end{array}$$

In formula (18), *M*_*j*_ refers to the arithmetic mean; *n* is the number of experts, and *C*_*ij*_ refers to the total number of scores of the *i*th expert on the *j* index.

The formula for calculating the full frequency is as the following formula:

(19) $$\begin{array}{c} K_{j}=\frac{m_{j}}{n_{j}}. \end{array}$$

In formula (19), *K*_*j*_ is the full frequency, and the value of *K*_*j*_ is between 0 and 1.

The formula for calculating the coefficient of variation is as the following formula:

(20) $$\begin{array}{c} V_{j}=\frac{P_{j}}{M_{j}}. \end{array}$$

### 3.3. Questionnaire Survey Method

A questionnaire was made for the basic situation of community residents, willingness to participate in physical exercise, consumption level of sports, exercise venues, exercise items, sports organization management, and fitness guidance in their own communities. A total of 500 questionnaires were distributed to the residents of 3 streets in the central district of the city. Starting from the needs of this research, after conducting interviews with relevant experts, two questionnaires were designed. The design of the questionnaire strictly followed the selection of basic principles and methods. Relevant survey questions for the current situation of sports public services were preliminarily designed. After asking the relevant experts to revise and improve the content of the questionnaire, the relevant questionnaires were distributed, and the distribution objects were the actual managers of community sports and the two groups of residents participating in it. The purpose of the questionnaire design research was to understand the development status of the validity test of the questionnaire: in order to ensure the scientificity and validity of the stable questionnaire survey in this study, the designed questionnaire was sent to 10 experts for expert logic test. It was necessary to test the validity of the questionnaire including content, structure, and overall aspects. The investigation materials were classified and coded, and Spss21 is used to analyze data. SPSS21 Chinese version is a very powerful professional data analysis tool. SPSS21 version is convenient and easy to use. It adopts the input method and the management module similar to the Excel table, and data statistics is also extremely simple and convenient.

## 4. Simulation Experiment Analysis on the Contradiction between Supply and Demand of Public Sports Services

In this study, the actual valid questionnaire data were collated, and the results are shown in Table 1.

The system of inter-class sports activities needs to be fully implemented. 25–30 minutes of large-scale inter-class sports activities should be arranged every morning, and students should be seriously organized to do radio gymnastics. The collective sports activities needs to be carried out: boarding schools should insist on doing morning exercises every day.  Table 2 shows the standards for sports venues in national and local compulsory education schools.

**Figure 3:**
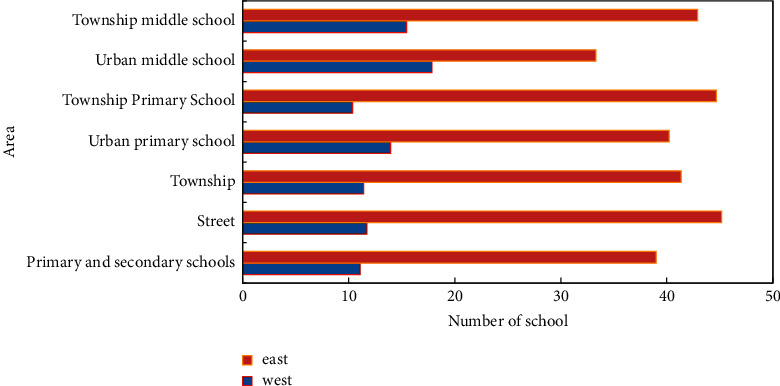
School situation in different regions.

A total of 25 primary schools and 25 secondary schools in four eastern and central provinces were investigated for school physical education in this study. The study period for primary and junior high schools in China's compulsory education stage is the 6–3 system, the 5–4 system, the 5–3 system, and the nine-year consistent system, respectively. This article still uses the name, junior high school, to refer to the original junior high school grades 7, 8, and 9 of the nine-year system. When the name of middle school is used in the following text, it refers to junior high school unless otherwise specified. The situation of schools in different regions is shown in Figure 3.

**Figure 4:**
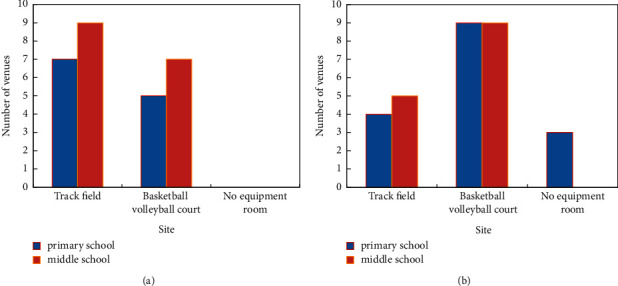
Sports venues and equipment in eastern and central regions. (a) Eastern region. (b) Central region.

Based on the “Construction Standard of Rural Ordinary Primary and Secondary Schools,” which is a lower standard for track field materials, and the standard that every 6 classes should have 1 basketball court or volleyball court, the current situation of school sports venues in various regions of China was investigated (the sports venues and equipment in the eastern region are shown in Figure 4(a)). In the eastern and central regions of this survey, the compliance rate of the middle school track and field is higher than that of primary schools, and secondary schools that fail to meet the standards are usually due to insufficient field area. Primary schools that do not meet the standards are mostly because there is no track and field at all. The central region is mainly underconstructed (the sports venues and equipment in the eastern region are shown in Figure 4(b)). Judging from the materials of the track and field, the plastic track in the eastern region has become more popular, while the central region is still dominated by the cinder track. In addition, 8 primary and secondary schools in the eastern region have indoor gymnasiums, 5 in the central region, and 4 schools in the eastern and central regions have swimming pools. Judging from the situation of school basketball volleyball court, the standard rate of middle school basketball volleyball court is also better than that of primary school, and the middle and primary schools are better than the eastern middle and primary schools. This is mainly because schools with insufficient track and field construction usually use simple basketball and volleyball courts as venues for school physical education and daily teaching activities. In addition to basketball and volleyball courts, the most common sports facilities in most schools are ping pong tables. Except for schools with extremely poor sports facilities, almost every school has a certain number of outdoor table tennis tables.

**Figure 5:**
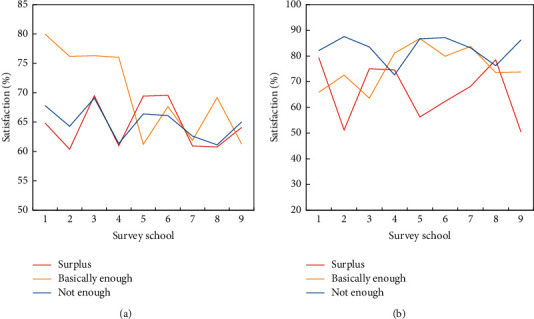
Sports equipment satisfaction. (a) Eastern region. (b) Central region.

The satisfaction with sports venues in middle schools is significantly higher than that in primary schools. The equipment satisfaction of the middle and primary schools in the eastern and central areas is similar, but the equipment satisfaction is slightly higher than the venue satisfaction (the sports equipment satisfaction survey in the eastern region is shown in Figure 5(a)). Sports equipment rooms are available in most schools. Although more than 70% of the schools in the equipment self-assessment believe that they have equipped sports equipment in accordance with the “Basic Standard for Trial Implementation of National School Sports Hygiene Conditions,” the proportion is obviously high from the survey and visit of some schools. The proportion of schools that can be fully configured according to this standard is not too high. Most schools can configure some frequently used sports equipment that is not easily damaged, and the easily worn sports equipment is rarely updated even if it has been configured (the satisfaction survey of sports equipment in the central region is shown in Figure 5(b)).

**Figure 6:**
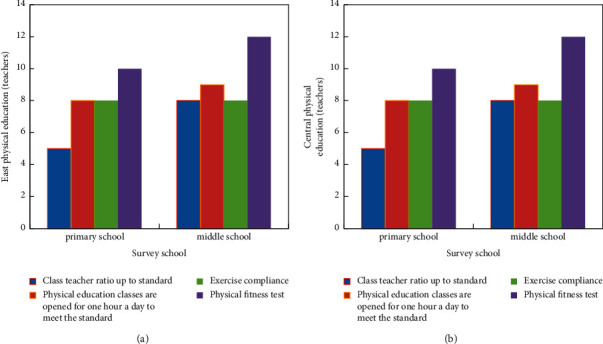
Physical education in primary and secondary schools. (a) Eastern PE teachers. (b) Central PE teachers.

The class-teacher ratio of primary and secondary schools is based on the standard of 6 classes with a full-time physical education teacher. Overall, only slightly more than half of the schools equipped with physical education teachers meet the standard. The allocation of PE teachers in middle schools is better than that in primary schools (The allocation of PE teachers in the east is shown in Figure 6(a)). Through the understanding of some eastern schools, some schools are more inclined to recruit main subject teachers although there is a shortage of physical education teachers. They usually let other teachers come to guest appearances in the case of insufficient physical education teachers. Therefore, the eastern school's emphasis on school physical education is not proportional to the development of education (the central PE teachers are equipped as shown in Figure 6(b)).

Taking 4 lessons per week for grades 1–2 in primary schools, 3 lessons per week for grades 3–6 and junior high schools, and 2 lessons per week for high schools, the survey found that the eastern region's physical education curriculum compliance rate was significantly higher than that in the central region. The situation of non-standard physical education in primary schools in the central region is relatively serious, especially the lack of physical education courses for grades 1–2 in primary schools. The arrangement of school morning exercise is related to the school accommodation system. Usually, the school with the accommodation system will carry out morning exercise activities, although during the implementation process, the morning exercise time becomes a signal for students to get up and prepare time before class. In the survey, most of the middle and primary schools in the Middle East could carry out and implement large-scale inter-class activities. From the perspective of the content of activities, they were mainly broadcast gymnastics (winter long-distance running). Some schools could carry out certain forms of sports activities, especially in the ninth grade. Because of the additional physical examination in the senior high school entrance examination, they paid more attention to quality practice during the large class. However, there were also some schools that did not implement large inter-class activities and only replace them with eye exercises. The extent of the development of large recesses is largely consistent with the conditions of school sports venues. The better the school venues and facilities are, the better the development of large recesses will be. Judging from the situation of extracurricular activities arranged by schools, most of the middle and primary schools in the east and middle could carry out extracurricular activities. In terms of self-assessment of one-hour daily exercise time in schools, exercise varies from region to region, this difference was more due to the difference in physical education. According to the survey, primary school students were better than middle school students in exercising for one hour a day. The main reason for this situation is the continuous increase of pressure for further education. The eastern primary and secondary schools could generally meet the standard of one physical fitness test per school year, and the physical fitness test in middle schools was also better than that in primary schools. Due to the incomplete data of each school on the pass rate, obesity rate, and myopia rate of middle school students who returned the questionnaire, it was impossible to analyze the students' physical condition through this survey. From the limited data, secondary school myopia and the primary school obesity rate were higher. The middle and primary schools in the east and middle were generally able to hold a track and field meeting every school year, and a relatively high proportion could hold 1–2 intramural ball sports competitions and 3–4 other sports competitions per school year. The ball games are mainly basketball, volleyball, and table tennis, and other sports competitions are mostly tug-of-war and rope skipping.

**Figure 7:**
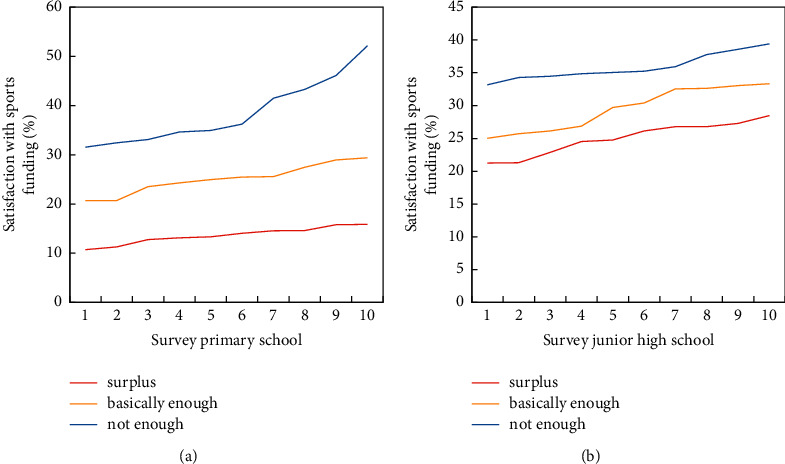
Results of the self-assessment survey on sports expenditures in primary and secondary schools in the eastern and central regions. (a) Elementary school. (b) Junior high school.

Several other important issues in school sports work include the budget expenditure of school sports funds, the acceptance of sports support from government departments, and the opening of school sports facilities. At present, China's primary and secondary education funds are mainly derived from public funds allocated according to the number of students in the school. It can be seen that the satisfaction degree of sports funds in primary and middle schools in the eastern and central regions is not high, and the satisfaction with sports funds in middle schools is higher than that in primary schools. Only 50% of schools choose sports funds that are basically sufficient. There is little difference in the satisfaction level of sports funding in middle schools in the eastern and central regions. The satisfaction with sports funding in primary schools in the eastern region is higher than that in the central region (primary school funding is shown in Figure 7(a)). Judging from the amount of sports expenditures filled in by some schools, 2000–3000 yuan per school year for primary schools and 4000–5000 yuan for middle schools are more common. Even if a school has 1,000 students, the per capita sports expenditure is less than 10 yuan. Some schools with sports teams have spent 10,000–20,000 yuan in sports funds. Even if it is all used for daily sports work, the funds are quite limited, not to mention training and competition. Education, sports, and other government departments sometimes provide certain sports services and support to primary and secondary schools, mainly in the form of sports equipment donations and business training, but the proportion and frequency are not high (The funding for middle schools is shown in Figure 7(b)).

The issue of the opening of school sports venues and facilities to the public has been widely concerned. Judging from the results of this survey, except for some schools in the middle and primary schools in the east and the middle, they do not have venues and facilities and are unable to open. Some schools are open because the sports facilities and teaching areas can be separated in the area, and some schools and public sports facilities are actually open. The most that other schools can do is to open them to their own school and surrounding students for free after school and during holidays. Under the encouragement of the local government's school opening policy and financial subsidies, some schools open some indoor venues for a fee, but the overall utilization rate is not high because the price is significantly higher than that of socially operated sports venues.

The selection of residents' sports activities and types of facilities is shown in Table 3. Residents ranked the top three purposes of participating in physical activity according to their importance. Maintaining good health is the first purpose of residents participating in sports activities, which is far ahead of other purposes, reaching 64%. Maintaining and improving the body is the second purpose of residents participating in sports activities, accounting for 44%, which is 6 percentage points higher than the relatively low goals of improving physical fitness and making friends at the same level. Recreation is the third purpose of residents participating in sports activities, accounting for 46%. However, from the further analysis of the average ranking, maintaining good health, improving physical fitness, and entertainment accounted for a relatively high proportion, ranking the top three. Except for the second main purpose, the other purposes are the same, indicating that the main purpose of residents participating in sports activities is consistent with the essential function of sports. Combining all the purposes of activities, it is found that the residents' sports interest has gradually shifted from direct interest to indirect interest, and the purpose of activities has diversified, which poses a challenge to the supply of community public sports facilities.

With the popularization of the concept of national fitness, people have gradually realized the role of physical fitness in life, but at the same time, the quality cannot be guaranteed from the perspective of supply side structural reform; this paper investigates the supply and demand of public sports services from the current situation, deeply analyzes the supply and demand contradiction of public sports services, defines the current supply and demand situation of public sports in China, uses the genetic algorithm to find out the contradiction between them, and puts forward the solution of marketization, socialization, organization, and legalization of public sports services.

Due to the different sports preferences of residents and the range of activities, the selection of public sports facilities is shown in Table 4. Through sorting and analyzing the top three important places where residents often participate in sports activities, it is helpful to determine the characteristics of residents participating in sports activities. In the first place, fitness paths accounted for 67%, far exceeding other sports venues. In the second place, outdoor fitness equipment and parks and squares accounted for 44%. In the third place, other types of sports venues accounted for 57%, which was mainly due to the diversity of residents' sports activities determined by the diversity of residents' sports venues. Secondly, the average ranking analysis of residents' top three sports venues show that outdoor fitness equipment, other sports venues, and chess and card venues are the top three residents' sports venues on average. It can be seen that there is a large discrepancy between the importance ranking and the average ranking, and the importance ranking and the average ranking of other sports venues are higher, indicating that community residents participate in various types of sports activities. However, the overall analysis of free venues provided by the government is still the main choice for residents to engage in sports activities. This has a certain relationship with the income of residents. Genetic algorithm can reasonably allocate the optimal activity type for residents according to the characteristics of residents' sports activities, the stadium, and the number of activities.

**Figure 8:**
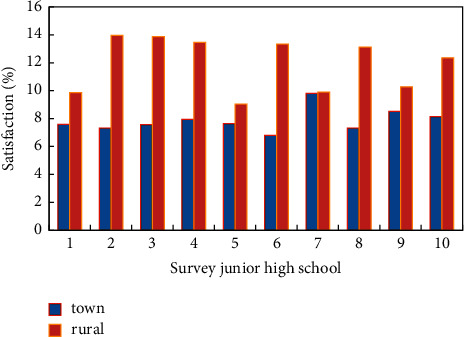
Comparison of sports venue equipment satisfaction in urban and rural areas.

Judging from the survey, the urban primary and secondary school track and field and basketball and volleyball courts are slightly better than the towns. The reasons for the great difference between school stadiums and gymnasiums are as follows: on the one hand, the streets are located in the urban area, there is no guarantee of constructing land for sports venues, so the sports venues cannot meet the standards. On the other hand, township schools, especially those where the local government is located, usually do not lag behind in the construction of school infrastructure, but the school sports facilities in rural areas outside the location of the township government are not guaranteed at all. Meanwhile, the vast majority of plastic track and field venues are located in urban primary and secondary schools. As far as the configuration of outdoor table tennis tables is concerned, there are slightly more schools in townships than schools in urban areas, and the shortage of land for schools in urban areas is one of the reasons. Another reason is that both urban schools and students have higher requirements for sports facilities than townships, so the construction of outdoor table tennis tables has gradually decreased. From the perspective of site satisfaction, township schools are generally more satisfied with sports venues than urban schools. Middle schools are more satisfied with sports venues than primary schools, and urban and townships have similar satisfaction with sports equipment. However, the level of satisfaction is higher than that of sports venues, and the reason why urban areas' satisfaction with sports venues and equipment is lower than that of townships is the shortage of school sports venues and equipment in urban areas. Another aspect is that physical education teachers in urban schools have higher requirements for sports venues and equipment than teachers in townships.  Figure 8 shows the comparison of the satisfaction of sports venues in urban and rural areas. At this time, according to the real-time evaluation characteristics of genetic algorithm, we need to timely find out the deficiencies of physical education teaching in urban and township primary and secondary schools, optimize physical education teaching resources, and promote the balanced development of public sports.

**Figure 9:**
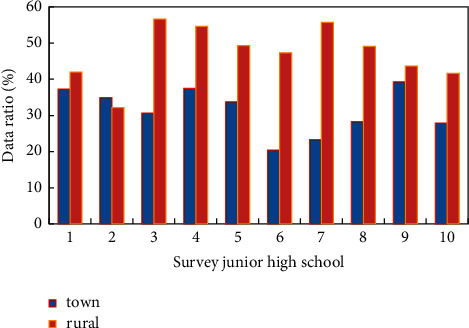
Results of a survey on physical education teaching in primary and secondary schools in urban and townships.

Figure 9 shows the survey results of physical education teaching in urban and township primary and secondary schools. From this survey, it can be seen that the class-teacher ratio in urban and township schools has not exceeded 60%. The lack of teachers in school physical education reflects that Chinese school physical education is in a low position in school teaching, and the reason why the class-teacher ratio in urban schools is not much different from that in township schools is similar to that of sports venues and facilities. The compliance rate of physical education courses in urban schools is significantly higher than that in township schools. The proportion of urban schools offering morning exercises is smaller than that of township schools, which is mainly due to the relatively few residential schools with convenient transportation in urban areas. The urban schools are better than the township schools in terms of large-scale inter-class activities, and the forms are also more diverse than the township schools. There is little difference between schools in urban and rural in terms of one-hour exercise time per day and the development of extracurricular activities in schools, but the overall compliance rate of one-hour exercise time per day is less than 60%. Judging from the physical fitness test arranged by the school, the implementation rate of physical fitness test in urban schools is better than that in townships, which is also reflected in the recovered data. The myopia rate in urban schools is up to 40%, and the obesity rate is up to 30%. Both urban and township schools are generally able to hold a track and field meeting every school year, and there is not much difference in the development of ball games and other sports competitions.

**Figure 10:**
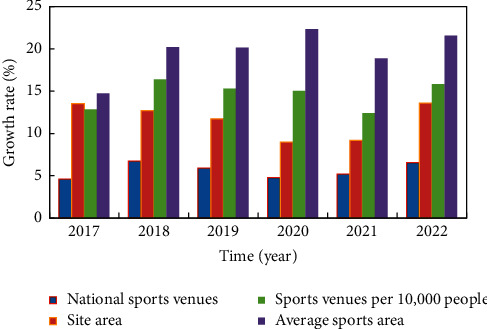
Comparison of main data of sports venues.

The main data comparison of sports venues is shown in Figure 10. Although the development achievements of China's sports public service are huge, so far, the current situation of China's sports public service is not very satisfactory. There are still two flaws: first, the total amount is insufficient. At present, the utilization structure of China's sports public facilities is extremely unreasonable. On the one hand, the number of stadiums serving competitive sports is huge, and there are tens of thousands of large stadiums for international and domestic competitions, which is second to none in the world. On the other hand, although the abovementioned data illustrating the achievements show that by the end of 2020, there will be 3.7134 million sports venues in the country, but the absolute number of sports venues managed by the education system, government agencies and institutions, and the military system, and the opening rate of these sports venues to the outside world is low.

## 5. Conclusion

In order to improve the utilization rate and service quality of public sports and balance the contradiction between supply and demand of public sports services, an optimal individual model of the genetic algorithm is established. Excellent individuals can spread rapidly in the area and converge to an optimal solution for the balance between supply and demand of public sports services in a relatively short period of time. At the same time, the algorithm proposed in this paper can effectively achieve the balance of public sports among multiple regions and can focus on optimizing service quality or operating cost according to actual needs. A major problem in the supply of China's sports public services is that the current China's sports public services are seriously insufficient in terms of resource supply. In addition, there is another problem that has attracted much attention in the development of China's sports public services, that is, the “relatively insufficient” supply still exists in regional imbalances and serious imbalances between urban and rural areas. Only by continuously expanding the scale and optimizing the structure in the process of supply, improving the availability and convenience of public sports services for the masses, and achieving the goal of full coupling between supply and demand can public sports serve the whole people in a true sense. The genetic algorithm proposed in this study helps to grasp the whole and each element of public sports service performance by deeply understanding the performance of each structural element of public sports service performance and to clarify the current problems and advantages of public sports service performance. This study makes a statistical analysis of the evaluation results of various structural elements of public sports services and compares the differences in the sub-performance of public sports services between regions through genetic algorithms, in order to promote the improvement and optimization of public sports services. Aiming at many problems existing in the sports public service and some action plans proposed by reference to national policies, this study analyzes the multiple dilemmas in optimizing the sports public service and tries to propose a feasible way to break through the dilemma. The collaborative innovation model of public sports service supply may be more inclined to economically developed regions. For remote and backward areas in China, due to the imperfect market and social organizations, the conditions for coordinated supply are not yet available, so the current development path should still be based on government support and supply. However, the coordinated supply of public sports services will be the overall trend in the future, and exploring the networked governance model will surely become an important way to carry it. Guided by the needs of residents' sports activities, aiming at the contradiction between supply and demand, a targeted strategy is proposed, and circular monitoring is achieved, so that the intelligent supply of public sports facilities can be realized, the sports interests of residents can be maximized and the informatization of government management can be achieved.

## Figures and Tables

**Table 1:** Arrangement of the actual valid questionnaire data.

Gender	Number of people	Percentage (%)
Male	250	55.6
Female	200	44.4
Total	450	100

**Table 2:** National and local compulsory education school sports venue standards.

Area	Types of school	Number of classes	Track field
National standard	Primary school	≤18	200 meters (ring) 1 piece
	Junior high school	≤18	300 meters (ring) 1 piece

National rural standard	Primary school	≤6	300 m–400 m (ring) 1 piece
	Junior high school	≤18	300 meters (ring) 1 piece

**Table 3:** Selection of resident sports activities and types of facilities.

Purpose of physical activity	1nd (%)	2nd (%)	3rd (%)
Stay healthy	64	26	4
Entertainment	23	26	46
Relax	31	36	31
Improve physical fitness	14	39	44

**Table 4:** Selection of public sports facilities.

Fitness venue	1nd (%)	2nd (%)	3rd (%)
Outdoor fitness equipment	11	44	42
Indoor gym	40	35	57
Outdoor fitness venues such as parks and squares	20	67	27
From home yard	27	23	45

## Data Availability

The data used to support the findings of this study are available from the corresponding author upon request.
